# Identifying adolescents at risk for suboptimal adherence to tuberculosis treatment: A prospective cohort study

**DOI:** 10.1371/journal.pgph.0002918

**Published:** 2024-02-27

**Authors:** Silvia S. Chiang, Joshua Ray Tanzer, Jeffrey R. Starke, Jennifer F. Friedman, Betsabe Roman Sinche, Katya León Ostos, Rosa Espinoza Meza, Elmer Altamirano, Catherine B. Beckhorn, Victoria E. Oliva Rapoport, Marco A. Tovar, Leonid Lecca

**Affiliations:** 1 Department of Pediatrics, Alpert Medical School of Brown University, Providence, Rhode Island, United States of America; 2 Center for International Health Research, Rhode Island Hospital, Providence, Rhode Island, United States of America; 3 Department of Pediatrics, Baylor College of Medicine, Houston, Texas, United States of America; 4 Section of Infectious Diseases, Texas Children’s Hospital, Houston, Texas, United States of America; 5 Socios En Salud Sucursal Perú, Lima, Peru; 6 Facultad de Ciencias de Salud, Escuela de Medicina, Universidad Peruana de Ciencias Aplicadas, Lima, Peu; St John’s National Academy of Health Sciences, INDIA

## Abstract

Adolescents account for an estimated 800,000 incident tuberculosis (TB) cases annually and are at risk for suboptimal adherence to TB treatment. Most studies of adolescent TB treatment adherence have used surveillance data with limited psychosocial information. This prospective cohort study aimed to identify risk factors for suboptimal adherence to rifampicin-susceptible TB treatment among adolescents (10–19 years old) in Lima, Peru. We collected psychosocial data using self-administered surveys and clinical data via medical record abstraction. Applying k-means cluster analysis, we grouped participants by psychosocial characteristics hypothesized to impact adherence. Then, we conducted mixed effects regression to compare suboptimal adherence–defined as <90% (missing >10% of doses)–between clusters. Treatment setting (facility vs. home) and drug formulation (single drug vs. fixed dose combination) were interaction terms. Of 249 participants, 90 (36.1%) were female. Median age was 17 (IQR: 15, 16.6) years. We identified three clusters–A, B, and C–of participants based on psychosocial characteristics. Cluster C had the lowest support from caregivers, other family members, and friends; had the weakest motivation to complete TB treatment; were least likely to live with their mothers; and had experienced the most childhood adversity. Among the 118 (47.4%) participants who received facility-based treatment with single drug formulations, adherence did not differ between Clusters A and B, but Cluster C had six-fold odds of suboptimal adherence compared to Cluster A. In Clusters B and C, adherence worsened over time, but only in Cluster C did mean adherence fall below 90% within six months. Our findings have implications for the care of adolescents with TB. When caring for adolescents with low social support and other risk factors, clinicians should take extra measures to reinforce adherence, such as identifying a community health worker or peer to provide treatment support. Implementing newly recommended shorter regimens also may facilitate adherence.

## Introduction

Globally, adolescents, defined by the World Health Organization (WHO) as people 10–19 years old, account for an estimated 800,000 incident TB cases per year [1,2]. In diverse settings, adolescents are at risk for suboptimal adherence to TB treatment, which includes missed doses and loss to follow-up [3–5]. Suboptimal treatment adherence leads to unfavorable outcomes for people with TB–specifically, increased antimicrobial resistance, treatment failure, and death–as well as for public health. Inadequate therapy leads to continued infectiousness and community transmission of *Mycobacterium tuberculosis*, particularly given adolescents’ many social contacts [6–8].

Among adolescents, age ≥15 years, male gender, prior TB illness, and coinfection with human immunodeficiency virus (HIV) have been associated with loss to follow-up from TB treatment [3,4,9,10]. However, because the existing literature on adolescent adherence to TB treatment has almost exclusively used surveillance data, the impact of more nuanced psychosocial and clinical factors–such as family difficulties, social support, stigma, mental health, satisfaction with healthcare services, adverse treatment events, and pill formulations–have not been evaluated. Moreover, most published studies on this topic have not examined missed doses as an outcome [3,4,9,10], yet missing >10% of doses is associated with unfavorable outcomes, including treatment failure, relapse, loss to follow-up, and death [11].

We carried out mixed-methods research to better understand adolescent adherence to treatment for rifampicin-susceptible TB. Prior to this current study, we conducted qualitative research consisting of in-depth interviews with 34 adolescents who were treated for TB, their primary caregivers, and 15 health providers [12]. We identified the following key barriers to adherence: the inconvenience of health facility-based directly observed therapy (DOT), which requires daily trips to the health center to take medication; long treatment duration; adverse treatment events; and symptom resolution. The support of adult caregivers was critical for helping adolescents overcome these barriers and remain motivated to complete treatment. We further observed that adolescents who were lost to follow-up or missed >20% of doses reported more childhood adversity, or adverse childhood experiences (ACEs), such as abuse, neglect, having divorced/separated parents, having an incarcerated family member, and household exposure to mental health problems, including substance use disorder [13].

Findings from our qualitative research generated hypotheses and informed the data collection tools for this prospective cohort study, which aimed to identify individual and regimen characteristics associated with adolescent adherence to TB treatment [12]. We hypothesized that adolescents with stronger social support and less childhood adversity would have better treatment adherence than those with weaker social support and more childhood adversity, and that adherence would decrease in later months of treatment.

## Methods

### Setting

This study took place in Lima, Peru and was conducted in partnership with Socios En Salud (SES; the Peruvian branch of Partners In Health). Peru is an upper middle-income country with an estimated TB incidence of 116 per 100,000 population [14]. Of people on TB treatment, 72% receive care at primary health centers run by the Ministry of Health [15]. In Peru, for people without HIV infection, treatment for drug-susceptible TB disease (other than miliary, central nervous system, or osteoarticular TB) consists of a two-month intensive phase with daily isoniazid, rifampicin, pyrazinamide, and ethambutol, followed by a four-month continuation phase with thrice weekly isoniazid and rifampicin. At the time of the study, the continuation phase was seven months of daily isoniazid and rifampicin for people living with HIV, and treatment for isoniazid-monoresistant TB (regardless of HIV status) was nine months of daily rifampicin, levofloxacin, and ethambutol, with the addition of pyrazinamide for the first two months [16]. Physicians may prescribe additional doses or daily dosing during the continuation phase for severe disease, slow treatment response, and/or suboptimal adherence. Of note, “daily” dosing in Peru is operationalized as six-days-a-week dosing since TB treatment is not administered on Sundays. Physicians are encouraged to prescribe fixed dose combination (FDC) pills, which combine the different medications, to reduce the pill burden compared to single drug formulations (SDFs); however, FDCs are not always available [17].

Data collection occurred from October 2020 to August 2022, at the height of the Covid-19 pandemic. Before the pandemic, Peru’s Ministry of Health mandated daily, facility-based directly observed therapy (DOT) under the supervision of providers–specifically, nurses or nurse technicians (the equivalent of licensed vocational nurses)–for the entire duration of TB treatment [16]. However, during the Covid-19 pandemic, after receiving facility-based DOT for the first two weeks of the regimen, selected adolescents were allowed to take treatment at home under the supervision of an adult caregiver, who signed an agreement with the health center assuming responsibility for supervising daily pill taking and ensuring adherence. Focus groups with TB treatment providers revealed that the selection of adolescents for home-based treatment depended on two factors: providers’ perceptions of the adolescent and their family’s level of responsibility, and the frequency and severity of adverse treatment events in the first two weeks of the regimen [18]. Every week, adolescents and/or their caregivers picked up TB medications from the health center, and caregivers reported treatment adherence to providers. Adolescents on home-based treatment with regular access to a smartphone received video-DOT in addition to caregiver supervision. Video-DOT consisted of taking their pills on video calls with providers or sending recorded videos of themselves taking their medication to providers via WhatsApp (Facebook, Inc.) [19]. On video calls and videos, adolescents demonstrated putting the correct pills in their mouth and swallowing them. Providers marked adolescents as adherent each day that a video call was completed or a video was received. Adolescents who were not selected for home-based treatment continued taking medication at the health center, where adherence was ascertained via direct observation of pill taking by providers.

Start and end dates of the intensive and continuation phases; dosing frequency; and numbers of completed and missed doses in each treatment month were recorded on DOT cards stored at the health center. For facility-based treatment and home-based treatment with video DOT, completed doses were marked each day that adolescents were observed taking their medication. For home-based treatment, self-reported adherence during the previous week was recorded at medication pickup.

### Study participants

We reviewed lists of people currently on TB treatment at 106 health centers and recruited all adolescents who were 10–19 years old at treatment initiation, diagnosed with TB disease of any anatomic site with or without microbiological confirmation, and had not yet started or were in the first four weeks of TB treatment. We provided sputum specimen testing using Xpert MTB/RIF Ultra (Cepheid, Sunnyvale, U.S.A.) to adolescents with pulmonary TB being considered for enrollment who had not yet received drug susceptibility testing of their *M*. *tuberculosis* strain. These adolescents produced sputum spontaneously. The study did not provide sputum induction, gastric aspiration, bronchoalveolar lavage, or sampling of extrapulmonary sites. We excluded adolescents with rifampicin-resistant TB confirmed by Xpert MTB/RIF, Xpert MTB/RIF Ultra, or GenoType MTBDRplus (Hain Lifescience, Nehren, Germany). Because we collected sensitive information–including feelings towards their caregivers, satisfaction with TB care, and drug use–via a self-administered survey (to increase reliability), we excluded adolescents who could not read.

### Data collection

Between the third and fifth weeks of treatment, participants completed a survey (**S1 Text**) on a tablet computer using the REDCap (Research Electronic Data Capture; Nashville, U.S.A.) platform in a private space at home or a nearby health facility. Authors KLO and REM remained nearby to answer any questions. The purpose of the survey was to capture demographic and socioemotional data that may impact adolescent adherence to TB treatment, as informed by our qualitative research [12]. The survey consisted of previously validated scales, such as the adverse childhood experiences (ACEs), Patient Health Questionnaire-9 (PHQ-9) depression screening scale, and the Alcohol Use Disorders Identification Test (AUDIT). The survey also included new scales and individual items, which were written, piloted, and validated as described in **S2 Text**.

Authors KLO and REM abstracted clinical variables, including treatment details, from each adolescent’s DOT card and medical chart. Data were entered on tablet computers using SEIS Datos (Socios En Salud Sucursal Perú; Lima, Peru).

### Clinical care

Participants received routine TB care from the Ministry of Health. The only health services provided by the study were (1) sputum analysis using Xpert MTB/RIF Ultra for those who had not yet had drug susceptibility testing (DST) at the time of study recruitment and (2) evaluation by a licensed psychologist employed by SES for those who had concerning PHQ-9 results (indicating moderate depression, severe depression, or suicidal ideation) or requested to speak with a psychologist for any reason. Participants whom the psychologist diagnosed with severe depression and/or had suicidal ideation were referred emergently to psychiatric care, and those with moderate depression received counseling. The psychological care algorithm and the numbers of participants who underwent evaluation can be found in **S1 Fig**.

### Ethics

The institutional review boards of Peru’s National Institute of Health and Rhode Island Hospital approved this study. Additionally, this study was approved by SES’s Community Advisory Committee (CAC), an independent group comprised of community members, including multiple individuals who previously had TB. The CAC reviews research projects and makes recommendations from the perspective of affected communities [20]. Written informed consent was obtained from participants ≥18 years old and parents/legal guardians of minors. Informed assent was obtained from participants <18 years old.

### Inclusivity in global research

Additional information regarding the ethical, cultural, and scientific considerations specific to inclusivity in global research is included in **S1 Checklist**.

### Outcome definitions

Our primary outcome was suboptimal dose-based adherence, defined as missing >10% of doses, which is associated with unfavorable treatment outcomes [11]. We calculated dose-based adherence for each regimen (some participants received more than one regimen) using the following formula and expressed as a percentage:

$$\left(1-\frac{number\;of\;doses\;missed}{number\;of\;total\;doses\;prescribed}\right)\times 100$$

In some health centers, monthly food baskets for people on TB treatment are withheld unless they achieve a certain level of adherence. Several TB treatment providers reported to our team that they did not mark missed doses to help adolescents receive their rations. Therefore, we did a sensitivity analysis using time-based adherence for each regimen, which captured the difference between the amount of time it should have taken to complete the entire regimen, had all prescribed doses been taken on time, versus the amount of time it actually took. We expressed time-based adherence as a percentage, calculated using the following formula:

$$\left(1-\frac{actual\;treatment\;duration\;in\;days-expected\;treatment\;duration\;in\;days}{expected\;treatment\;duration\;in\;days}\right)\times 100$$

Actual treatment duration was calculated as the sum of the numbers of days of the intensive and continuation phases. To calculate the duration of each treatment phase, we subtracted its start date from its end date. We considered these dates to be reliable as they were abstracted from each adolescent’s DOT card and corresponded with the automated dates of routine chest radiographs obtained before treatment, at the end of the intensive phase, and at the end of the continuation phase. Expected treatment duration was calculated using the following formulas: $\frac{number\;of\;prescribed\;doses}{6}\times 7\;$ for daily dosing (i.e., six days per week since no doses were given on Sundays), and $\frac{number\;of\;prescribed\;doses}{3}\times 7$ for thrice weekly dosing.

Both dose-based and time-based adherence were set to 0% for the participants who were lost to follow-up from treatment.

### Statistical analysis

The aim of this analysis was to evaluate the relationship between participant characteristics, treatment variables, and regimen adherence. We conducted k-means cluster analysis using the *kamila* package to categorize participants by the following demographic, social, and clinical variables previously shown to impact treatment adherence: age, gender, childhood adversity, accompaniment to the health center, adolescent’s relationship with their caregiver, social support, commute to the health center, satisfaction with healthcare services, treatment self-efficacy, and frequency of adverse treatment events, and substance use [21]. We used cluster analysis to characterize participants with respect to these variables while avoiding the problem of multicollinearity in regression, as we anticipated that many of the measured traits of personal circumstance would share common causes (e.g., family dysfunction causing childhood adversity, low levels of social support, and depression). Cluster analysis can help identify similar groups of participants with fidelity to the holistic interactions between aspects of personal circumstance. Interpretation of the clinical significance of clusters identified empirically can facilitate a more parsimonious analysis plan. To select the number of clusters, we used an iterative process, specifying different numbers from 5 to 2 and examining intercluster descriptive statistics until we arrived at a clinically meaningful number of clusters.

Next, we used mixed effects regression to evaluate the odds of suboptimal adherence based on cluster. We included main effects and interactions, modeling adherence regressed on the cluster, treatment setting (facility vs. home), and drug formulation (SDF vs. FDC). Our regression model did not include daily dosing in the continuation phase or the use of second-line medications due to the small numbers of regimens with these characteristics (27 and 17 regimens, respectively). We introduced random effects for the individual and the health center where they received TB care to avoid violating the assumption of independence.

Additionally, using mixed effects regression with random effects for individual and health center, we evaluated changes in time-based adherence across treatment months 1–12, representing the range of regimen durations in the study. We allowed for separate rates of change for participant characteristics by including as cluster as an interaction term.

The median percent of missing values per variable was 0.4% (interquartile range (IQR): 0–1.2%). Therefore, we used the *mice* package to impute missing data where they occurred [22]. We used regression to calculate p-values. All analyses were conducted with R (R Foundation for Statistical Computing, Vienna, Austria, 2022).

### Sample size and power

Initially, we planned a sample size of 400 based on data suggesting that 20% of participants would have suboptimal treatment adherence and a commonly used guideline of ten outcome events per independent variable [23,24]. This sample size would have allowed us to consider eight variables in our regression model. However, due to higher project costs and less access to potential participants during the COVID-19 pandemic, we were unable to achieve our original sample size. We performed a secondary power calculation based on the clusters identified in our actual sample. To estimate a lower limit on power, we considered the effect size that we would be able to detect between the two smallest clusters with 80% power. Again, assuming that 20% of participants would have suboptimal treatment adherence, we estimated that we would be able to detect odds ratios ≥2.50 between the smallest clusters at a significance level of 0.05. **S3 Text** contains additional details and explanation.

## Results

**Fig 1:**
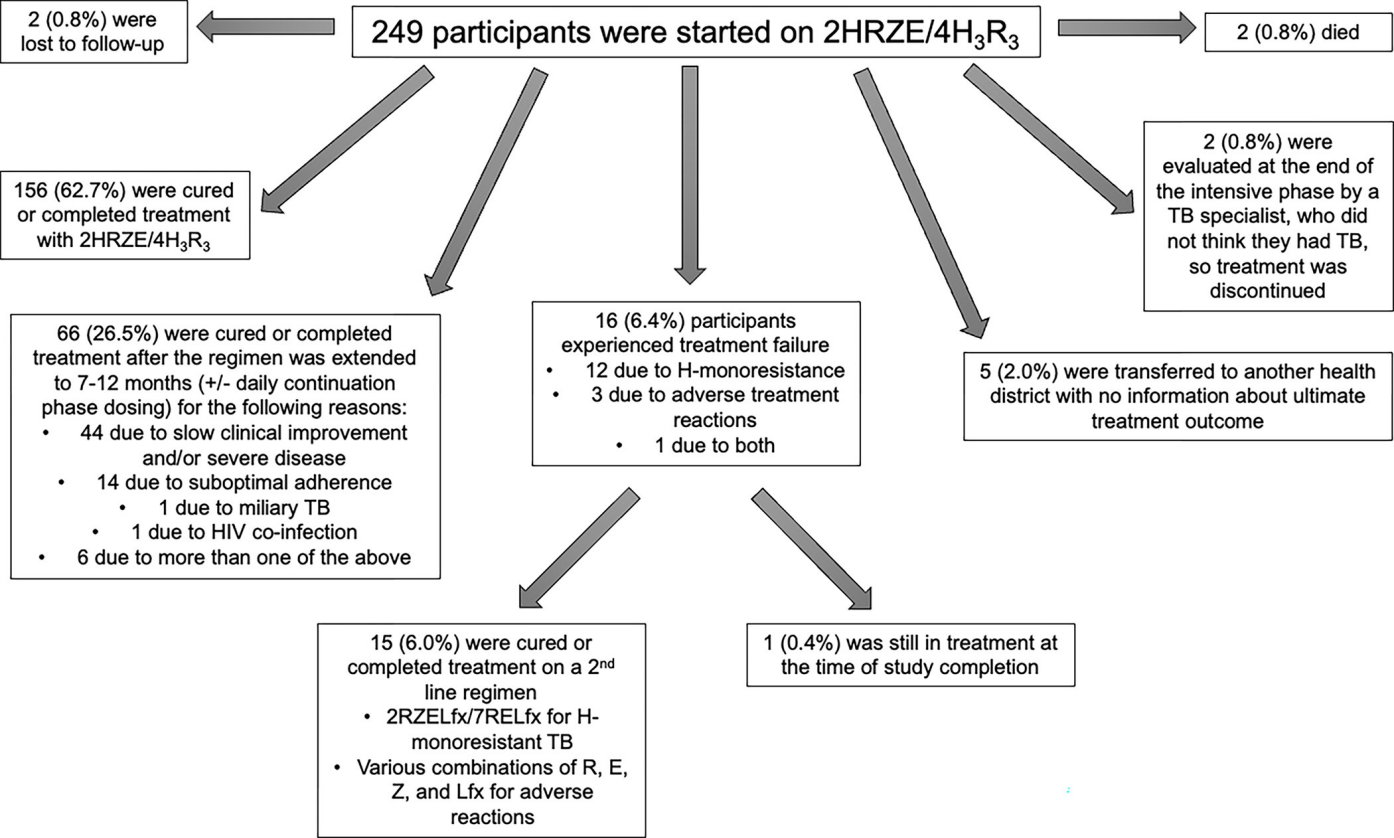
Treatment outcomes of the 249 participants. Abbreviations: E, ethambutol; H, isoniazid; HIV, human immunodeficiency virus; Lfx, levofloxacin; R, rifampicin; TB, tuberculosis; Z, pyrazinamide; 2HRZE/4H_3_R_3_, 2 months of HRZE followed by 4 months of HR thrice weekly; 2RZELfx/7RELfx, 2 months of RZELfx followed by 7 months of RELfx.

We invited 301 eligible adolescents to participate in the study; 46 (15.3%) did not enroll because they or their parents did not wish for them to participate, and three (1.0%) did not enroll because did not have a parent or legal guardian available to sign informed consent. Of 252 (82.1%) participants who enrolled in the study, two withdrew, and one declined to complete the survey. Of the remaining 249 participants, 90 (36.1%) were female, and the median age was 17 (IQR: 15–18) years. Three (1.2%) participants had been treated previously for TB disease. No participants had received treatment for *M*. *tuberculosis* infection, also known as tuberculosis preventive therapy (TPT), which is recommended for household contacts of people with TB and other individuals at risk for TB in whom TB disease has been ruled out [25]. One (0.4%) participant was living with HIV. Among the participants, 220 (88.4%) had pulmonary TB, 26 (10.4%) had extrapulmonary TB, and 3 (1.2%) had both pulmonary and extrapulmonary TB. Participants had the following types of extrapulmonary TB: pleural (n = 16), peripheral lymph node (n = 8), abdominal (n = 2), mastitis (n = 1), miliary (n = 1), and cutaneous (n = 1). **Fig 1** illustrates the treatment trajectories of the participants.

Twenty-six participants received daily dosing in the continuation phase: one for TB/HIV co-infection, one for miliary TB, 13 for isoniazid-monoresistant TB, and 11 for unspecified reasons.

The 249 study participants received 266 regimens in 71 different health centers across Lima. Eight-six (32.3%) regimens included FDCs. One hundred sixty-four (61.7%) regimens were administered entirely at the health facility, while the remainder were administered at home: 85 (34.1%) under family supervision only, and 17 (6.4%) using video DOT (**S1 Table**).

### Clusters

**Fig 2:**
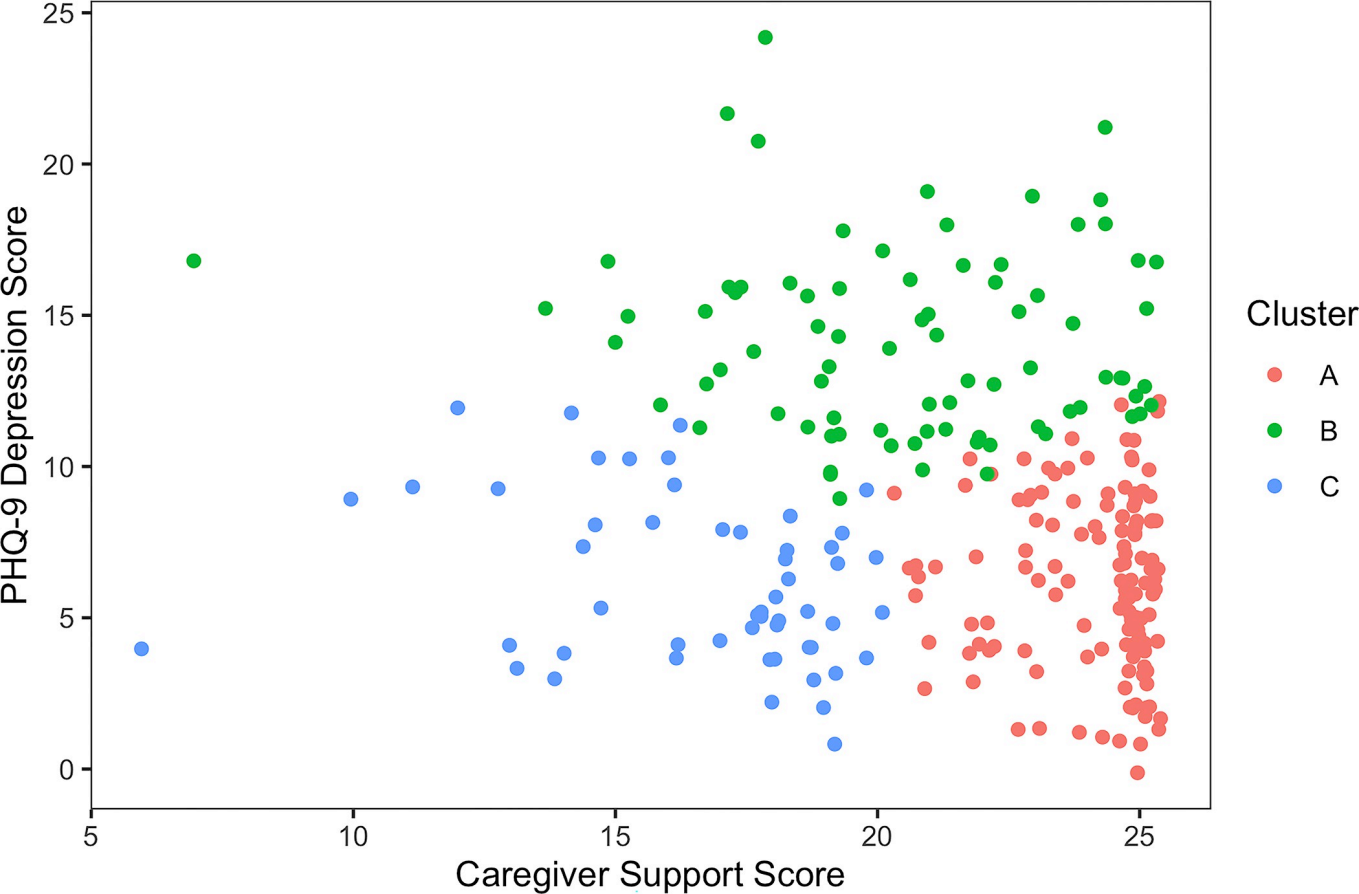
Clustering of participants*. *Caregiver support (measured by a new scale; range: 5–25) and depression (measured by the PHQ-9; range: 0–27) were selected to illustrate clustering because these were the variables by which the clusters differed the most.

We identified three clusters of participants with distinct profiles (**Table 1, Fig 2**): A (116 participants, 124 regimens), B (83 participants, 90 regimens), and C (50 participants, 52 regimens). Cluster A participants had the highest levels of support from their caregivers (including accompaniment to the health center), friends, and other family members; lowest ACE scores of all three groups; highest frequency of obeying caregivers; strongest motivation to complete treatment; highest satisfaction with health care; and shortest commute to the health center. A large proportion of Cluster B participants was female compared to Clusters A and C. Participants in cluster B were more likely to experience depression, anticipated stigma at being seen receiving TB care, and adverse treatment events in the first month of therapy. Cluster C participants were the least likely to live with their mother and had the highest ACE scores. Cluster C also had the lowest levels of social support, motivation to complete TB treatment, and satisfaction with TB care. Participants in Cluster C were least often accompanied to the health center by a caregiver or other family member. The clusters did not differ with respect to worry about Covid-19 transmission at or on the way to the health center (**S2 Table**).

**Table 1:** Variables used in cluster analysis.

n (%) or median (IQR)	Cluster A (n = 116)	Cluster B (n = 83)	Cluster C (n = 50)	p-Value*
Male gender	81 (69.8)	45 (54.2)	33 (66.0)	0.07
Age (years)	17.0 (15.0, 18.0)	16.0 (15.0, 18.0)	17.0 (15.3, 19.0)	0.10
Travel time to health center (minutes)^&^	9 (3, 9)	9 (9, 9)	9 (9, 9)	0.003
Walks/bikes to health center (vs. takes taxi/motor-taxi/bus)	66 (56.9)	45 (54.2)	25 (50.0)	0.71
Frequency of hunger due to poverty (days/week)^&^	0 (0, 1.8)	1.8 (0, 1.8)	1.8 (0, 3.5)	0.38
ACE score: 0 (lowest) to 10 (highest)	1 (0, 2)	2 (1, 4)	2 (1, 5)	<0.001
Lives with mother	104 (89.7)	72 (86.7)	36 (72.0)	0.01
Caregiver support: 5 (least) to 25 (most)	25.0 (23.0, 25.0)	21.0 (19.0, 23.5)	17.0 (15.0, 18.8)	<0.001
Obeys caregiver: 1 (never) to 5 (always)	5.0 (4.0, 5.0)	4.0 (3.0, 5.0)	4.0 (3.0, 4.8)	<0.001
Support from friends: 1 (least) to 5 (most)	5.0 (3.0, 5.0)	4.0 (2.0, 5.0)	2.0 (1.0, 4.0)	<0.001
Support from other family members: 1 (least) to 5 (most)	5.0 (4.0, 5.0)	4.0 (3.0, 5.0)	3.0 (2.0, 4.0)	<0.001
Frequency of being accompanied to health center: 1 (never) to 5 (always)	4.0 (2.0, 5.0)	4.0 (2.0, 5.0)	2.5 (1.0, 5.0)	0.004
PHQ-9 depression score: 0 (lowest) to 27 (highest)	6.0 (4.0, 8.0)	13.0 (11.0, 16.0)	7.0 (4.0, 9.0)	<0.001
AUDIT score: 0 (lowest) to 40 (highest)	0.0 (0.0, 0.0)	0.0 (0.0, 0.0)	0.0 (0.0, 0.0)	0.03^#^
Illicit drug use in last 12 months	14 (12.1)	10 (12.0)	11 (22.0)	0.20
Motivation score: 4 (least) to 20 (most)	20.0 (19.0, 20.0)	20.0 (18.5, 20.0)	17.5 (16.0, 20.0)	<0.001
Anticipated stigma of seen receiving TB care: 1 (never) to 5 (always)	1.0 (1.0, 2.0)	1.0 (1.0, 3.0)	1.0 (1.0, 2.0)	0.02
Frequency of adverse treatment events (days/week)^&^	0.0 (0.0, 1.8)	1.8 (1.8, 3.5)	0.0 (0.0, 1.8)	<0.001
Satisfaction with TB care: 4 (least) to 20 (most)	16.0 (16.0, 17.0)	16.0 (15.0, 17.0)	16.0 (15.0, 16.0)	0.02
Treatment self-efficacy: 4 (lowest) to 20 (highest)	11.0 (11.0, 12.0)	11.0 (10.0, 12.0)	11.5 (11.0, 12.0)	0.21

Abbreviations: ACE, adverse childhood experience; AUDIT, Alcohol Use Disorders Identification Test; FDC, fixed-dose combination; PHQ-9 (Patient Health Questionnaire-9); TB, tuberculosis.

*Reported p-values tested the assumption that the reported statistics (median or percentage) were equivalent between all three clusters. Because the groups were determined empirically by cluster analysis, we did not have an *a priori* hypothesis as to which groups would have higher or lower levels of each variable. This column is provided for the specific purpose of identifying which traits tended toward the largest differences between groups.

^&^In the original survey (see S1 Text), travel time to health center, the frequency of hunger, and the frequency of adverse treatment events were categorial variables, with response options given as fixed ranges. The only response option that did not include a fixed range was >2 hours for travel time to health center, but no participants selected this option. To facilitate statistical analysis, we converted these variables from categorical to continuous by replacing each range with its midpoint value. For example, 0–4 minutes (the first option for travel time to health center) was replaced by 2 minutes.

^#^0 (0.0%), 3 (3.6%), and 3 (6.0%) of participants in Clusters A, B, and C, respectively, were diagnosed with harmful drinking using the AUDIT.

### Adherence

**Fig 3:**
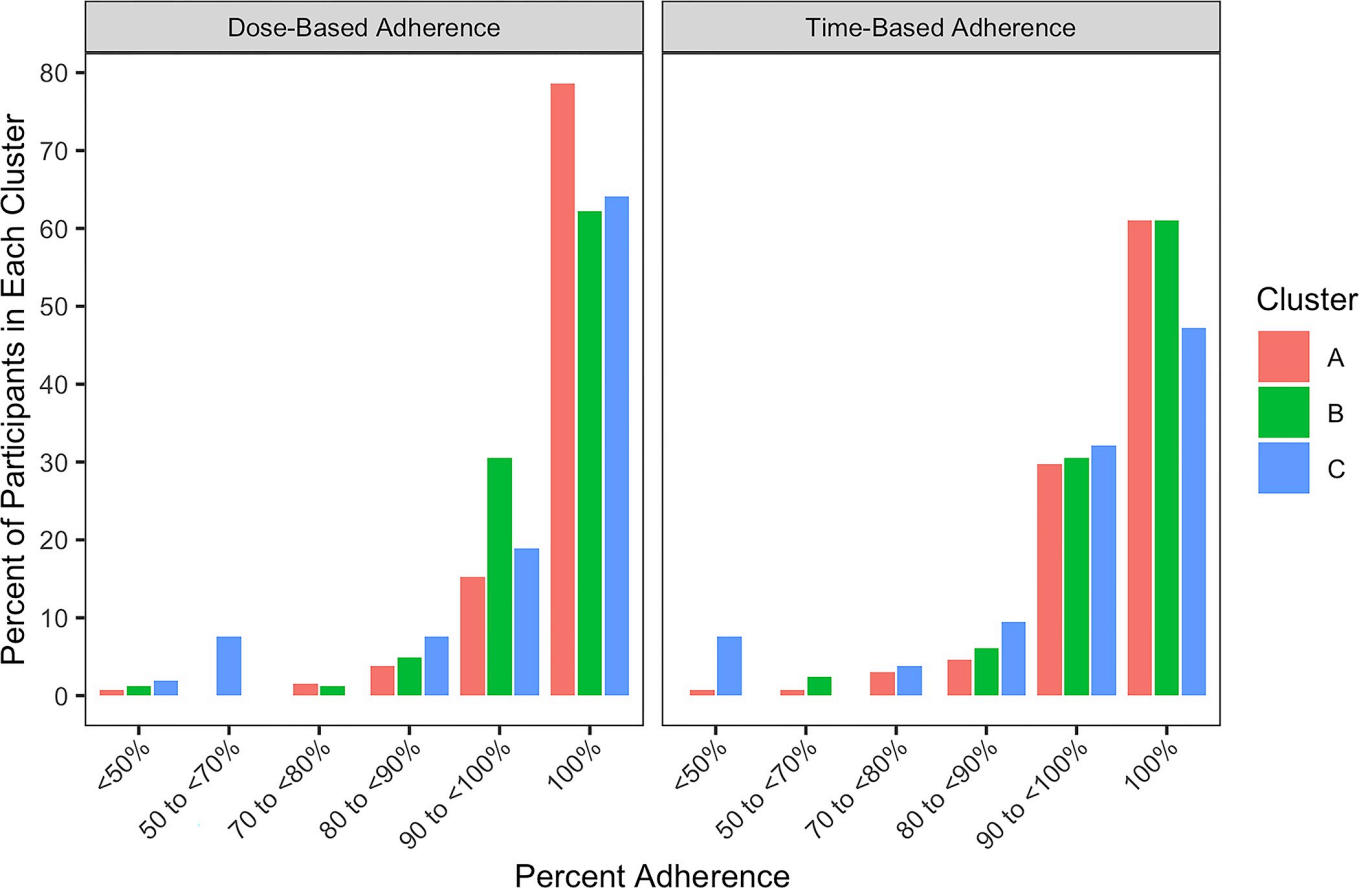
Distribution of adherence by cluster.

**Fig 3** illustrates dose-based and time-adherence by cluster. Among participants who received facility-based treatment with SDFs, Clusters A and B had similar adherence, but Cluster C participants had nearly six times as large odds of suboptimal adherence compared to Cluster A participants (**Tables 2 and S3**). We did not observe a difference in adherence by cluster for any of the other strata (based on treatment setting and drug formulation), but these analyses were limited by small group sizes. Sensitivity analysis did not change the interpretation of the findings (**S4 and S5 Tables**).

**Table 2:** Dose-based adherence, stratified by treatment setting and drug formulation.

Cluster	Total regimens (N)	Percent of regimens with suboptimal adherence (95% CI)	OR (95% CI) of suboptimal adherence*	p-Value
Facility-based, single-drug formulation				
A	53	5.7 (1.8, 16.1)	Ref	Ref
B	38	10.5 (4.0, 24.9)	1.96 (0.41, 9.32)	0.40
C	27	25.9 (12.9, 45.3)	5.83 (1.37, 24.83)	0.02
Facility-based, fixed-dose combination				
A	24	12.5 (4.1, 32.4)	Ref	Ref
B	17	5.9 (0.8, 32.0)	0.44 (0.04, 4.61)	0.49
C	5	20.0 (2.7, 69.1)	1.75 (0.14, 21.39)	0.66
Home-based, single-drug formulation				
A	31	3.2 (0.5, 19.6)	Ref	Ref
B	19	0 (0, 0)^#^	NA^#^	NA^#^
C	12	8.3 (1.2, 41.3)	2.73 (0.16, 47.46)	0.49
Home-based, fixed-dose combination				
A	16	6.3 (0.9, 33.5)	Ref	Ref
B	16	0 (0, 0)^#^	NA^#^	NA^#^
C	8	12.5 (1.7, 53.7)	2.14 (0.12, 39.47)	0.61

*Model contained drug formulation (single drug formulation vs. fixed dose combination) and treatment setting (facility-based vs. home-based) as interaction terms.

^#^Unable to estimate due to 0 observations of suboptimal adherence in that group.

Abbreviations: CI, confidence interval; NA, not applicable; OR, odds ratio.

**Fig 4:**
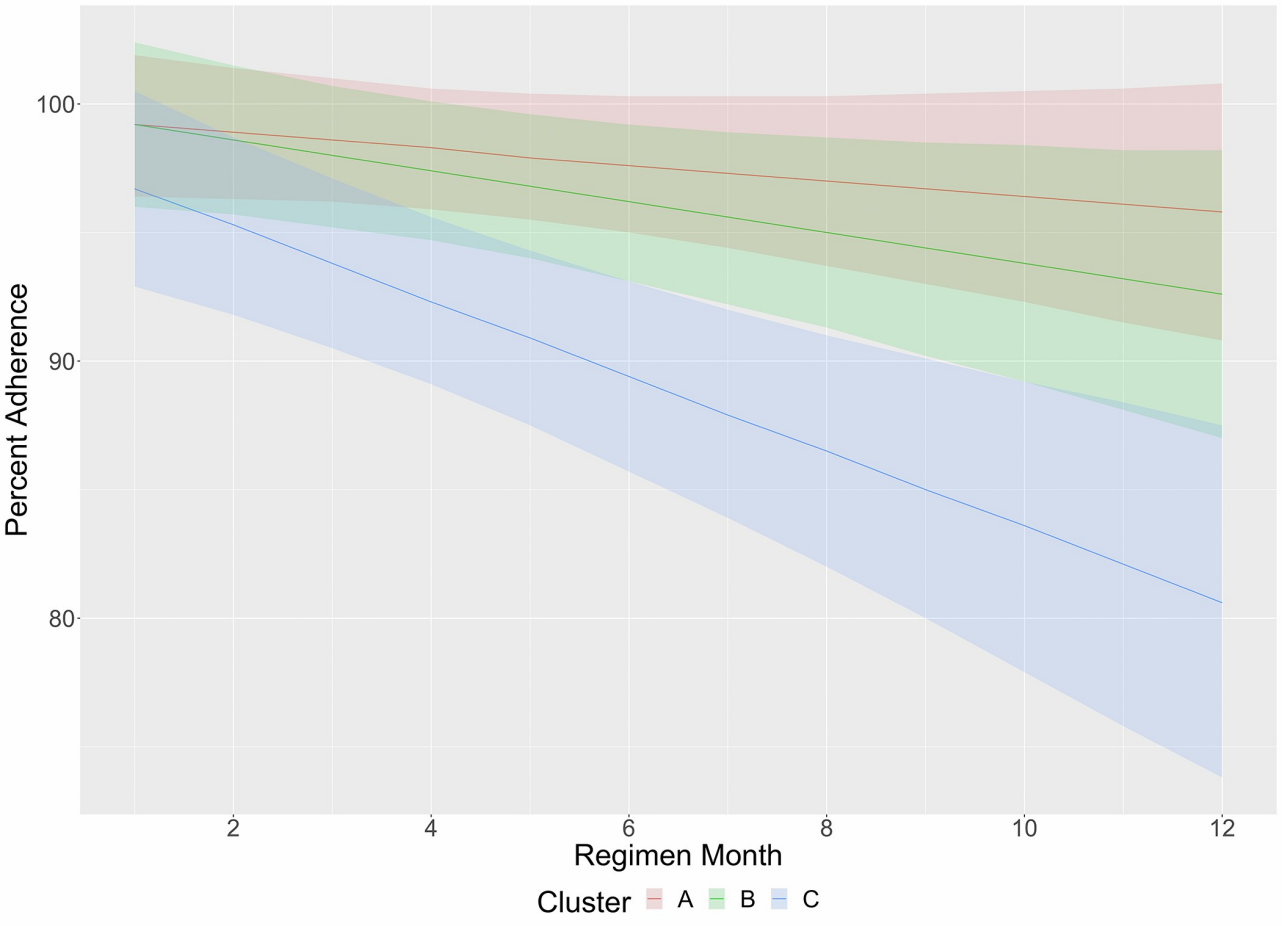
Dose-based adherence by regimen month, stratified by cluster.

Mean adherence (percent of doses taken on time) decreased throughout the course of treatment for Clusters B and C, but did not significantly change for Cluster A (**Fig 4**). For Cluster B, compared to mean adherence in month 1 (99.2%, CI: 96.2–102.2%), mean adherence was significantly lower beginning in month 8 (95.6%, CI: 92.1–99.1%, p = 0.04). For Cluster C, compared to mean adherence in month 1 (96.3%, CI: 92.3–100.3%), adherence was significantly lower beginning in month 4 (90.7%, CI: 87.1–94.2%, p = 0.02). By month 12, mean adherence was 80.7% (95% CI: 73.5–87.9%, p < 0.001).

## Discussion

In this study, we sought to understand the impact of family, social, and psychological circumstances on TB treatment adherence. We accomplished this aim using cluster analysis methodology, which identified three adolescent profiles based on traits shown to influence TB treatment adherence in our previously published qualitative research [12]. Participants in Cluster A–the group with the highest adherence throughout the entirety of treatment–most frequently were accompanied to the health center, live with their mother, and obey their caregiver. They also had the highest levels of social support from caregivers, other family members, and friends; motivation to finish treatment; and satisfaction with TB care. Participants in Cluster C–the group with the worst adherence–were least likely to demonstrate these characteristics and most likely to have experienced more childhood adversity. These observations support our primary hypothesis, which stemmed from our qualitative research findings [12]: adolescents with strong support would demonstrate the best treatment adherence, and adolescents with weak social support and a history of childhood adversity would demonstrate the worst.

We found partial evidence for our second hypothesis, that treatment adherence would decrease in later months of treatment. In Cluster A, adherence did not differ by treatment month, whereas in Clusters B and C, adherence decreased as treatment duration became longer. These findings also mirror our qualitative research conclusion that treatment fatigue is challenging for adolescents with all degrees of adherence, but those with good social support and motivation overcome these barriers [12].

The consistency of the findings between our cohort and qualitative studies, which were conducted among different groups of adolescents in Lima several years apart, strengthen the validity of the results. Similar observations have been reported from other research: TB treatment adherence among older children and adolescents in Mexico was associated with positive family dynamics [26], and a qualitative study in Lima found that caregiver support is vital to adherence to antiretroviral therapy among adolescents living with HIV [27].

This cohort had surprisingly high treatment adherence overall. In a retrospective cohort study of 249 adolescents with drug-susceptible TB in Lima between 2018–2019, we observed that 5.6% were lost to follow-up and another 14.9% completed <90% of doses on time [23]. There are two likely explanations for the high observed adherence in the current cohort. First, TB treatment adherence in Peru improved during the Covid-19 pandemic. According to surveillance data from the Ministry of Health, loss to follow-up declined among people of all ages with drug-susceptible TB, from 7.2% in 2018 to 4.8% in 2020 [28]. Programmatic data on missed doses are not available. The reasons for improved adherence to TB treatment during the pandemic are unknown; however, it is possible that home-based treatment facilitated adherence and, for adolescents assigned facility-based treatment, social distancing led to fewer activities (i.e., classes, social outings) that conflicted with health center visits. Second, there may have been a selection bias, as 16% of eligible adolescents were not enrolled in the study because they did not wish to participate, their parents did not want them to participate, they did not have a parent or legal guardian available to sign informed consent, or they could not read. These unenrolled adolescents may have been at higher risk for suboptimal adherence.

This study had additional limitations. We were unable to assess inter-cluster differences in adherence for participants who received home-based treatment or FDCs because these subgroups were small. Moreover, the allocation of treatment setting was not random, and, for the 68 participants who received neither in-person nor video DOT, adherence was self-reported. Additionally, a higher proportion of Cluster B received mental health care compared to the other two groups, and we cannot discard the possibility that this intervention may have impacted adherence. Next, the excluded adolescents who could not read or whose parents or guardians did not sign informed consent (because they were unavailable or disagreed with study participation) may have been similar to Cluster C participants with respect to family background, including level of caregiver support. Thus, their exclusion may have led to an underestimation of the association between the Cluster C profile and treatment adherence. Finally, we cannot exclude the possibility of inaccurate measurement of adherence. For adolescents who received home-based treatment under caregiver supervision without video-DOT, accurate reporting of adherence depended solely on the caregiver. Although the validity of caregiver reports was not ascertained, caregivers may have been less likely than the adolescents themselves to conceal missed doses (i.e., less social desirability bias), and the weekly reporting intervals may have reduced recall bias. Even for adolescents who received video-DOT or facility-based DOT, providers may not have accurately recorded adherence on DOT cards. As explained above, some providers caring for participants enrolled in this study reported not marking missed doses to help adolescents receive their food baskets, which were withheld from people with suboptimal adherence. For this reason, we conducted a sensitivity analysis using time-based adherence, which did not change the interpretation of the results.

Despite these limitations, the findings are highly consistent with those of our qualitative study conducted before the pandemic, thus strengthening their credibility. Our work highlights opportunities to improve adolescent adherence to TB treatment. In Peru, people diagnosed with TB are routinely evaluated by a social worker at the start of treatment. During this evaluation, the social worker could apply the ACEs scale as a surrogate for family dysfunction, and could ask adolescents to what extent they feel supported by caregivers, other family members, and friends. Adolescents who have family challenges and/or lack sufficient support from family and friends may benefit from peer accompaniment–a strategy that is effective for improving adolescent adherence to treatment for other diseases, including HIV and diabetes [29,30]. A pilot study conducted in Lima demonstrated the acceptability and feasibility of in-person peer support groups and group video-DOT (i.e., multiple adolescents taking their treatment together over a group video call) for adolescents living with HIV [31,32]. Notably, video-DOT for TB treatment is accepted by the WHO and the U.S. Centers for Disease Control and Prevention as a treatment delivery strategy because it achieves treatment outcomes that are at least as effective as conventional DOT, though these effectiveness data come mostly from high-income settings [33,34]. Nonetheless, growing evidence from high TB-burden, resource-limited settings like India also support the acceptability and feasibility of video-DOT [35,36]. Finally, rollout of two newly recommended shorter, four-month regimens for drug-susceptible pulmonary TB–the SHINE regimen for people <16 years old with non-severe disease and the rifapentine-moxifloxacin regimen for people ≥12 years old with any disease severity [37–39]–may decrease treatment fatigue. These interventions should be evaluated in adolescents with TB.

## Supporting information

S1 Checklist Inclusivity in global research. (DOCX)

S1 Fig Psychological care algorithm. (DOCX)

S1 Table Demographic, psychosocial, and clinical characteristics, stratified by treatment setting. (DOCX)

S2 Table Covid-related variables, stratified by cluster. (DOCX)

S3 Table Full regression output for suboptimal dose-based adherence. (DOCX)

S4 Table Time-based adherence, stratified by treatment setting and drug formulation. (DOCX)

S5 Table Full regression output for suboptimal time-based adherence. (DOCX)

S1 Text English translation of the survey. (DOCX)

S2 Text Survey development, including scale validation. (DOCX)

S3 Text Sample size and power. (DOCX)
