# Supplementary material for: Identifying adolescents at risk for suboptimal adherence to tuberculosis treatment: A prospective cohort study
Source: PLOS Glob Public Health. 2024 Feb 27;4(2):e0002918. doi: 10.1371/journal.pgph.0002918 (PMC10898721; doi:10.1371/journal.pgph.0002918)
Supplement: S1 Checklist — (DOCX) [file pgph.0002918.s001.docx]

**S1 Checklist: Inclusivity in global research**

**Ethical considerations, permits and authorship**

Provide details as to who granted permissions and/or consent for the study to take place in the Methods section of your manuscript. This should include the names of **all** ethics boards, governmental organizations, community leaders or other bodies that provided approval for the study. If individuals provided approval refer to these people by their role or title but do not list their name(s).

Reported on page number: 11

If there were any deviations from the study protocol after approval was obtained, please provide details of these changes in the Methods section of your manuscript.

Reported on page number: NA. There were no protocol deviations.

Did this study involve local collaborators that are residents of the country where the research was conducted or members of the community studied? If you do not have any authors from said communities, please provide an explanation for this below.

Yes. Six of the 12 authors – B. Roman Sinche, K. León Ostos, R. Espinoza Meza, E. Altamirano, M. A. Tovar, and L. Lecca – are Peruvian. L. Lecca is the senior author.

**Human subjects research**

Did you obtain written informed consent from a representative of the local community or region before the research took place? How did you establish who speaks for the community? Details of written informed consent obtained from study participants should be reported separately in the Methods section of your manuscript.

This study was conceptualized and carried out through a long-standing collaboration between the U.S.-based PI, Dr. Silvia Chiang, and her long-standing (>10 years) Peruvian collaborators. The research institution in Lima (the site of the research), Socios En Salud (SES), is affiliated with Partners In Health, based in Boston, U.S.A., but the directors and staff are all Peruvian. For nearly 30 years, SES has supported the Peruvian Ministry of Health in their efforts to fight tuberculosis and HIV/AIDS. SES has a Community Advisory Committee (CAC), which is comprised by lay people from high TB-burden communities within Lima where this study took place. The CAC includes people previously living with tuberculosis. The CAC approved this study. Additionally, this study was approved by the IRB of the Peruvian National Institute of Health and all health centers where recruitment took place.

How did members of the local community provide input on the aims of the research investigation, its methodology, and its anticipated outcome(s)?

The CAC, described above, provided input on the protocol, which was modified per their recommendations. Additionally, this research had to be approved by the health centers where participant recruitment took place. Requested changes from the health center staff also were incorporated.

When engaging with the local community, how did you ensure that the informed consent documents and other materials could be understood by local stakeholders?

SES has over 25 years of experience conducting research in high-TB burden areas within Lima. The consent forms and assent forms used in this study were based on forms used in multiple prior studies; thus, similar language was used. These and similar consent and assent forms have been used in studies conducted by SES for nearly 25 years. Moreover, in addition to providing the CAC and health center staffs with copies of the study protocol and consent and assent forms, a Peruvian study coordinator prepares slides to introduce the study using lay terminology. The study coordinator checks for understanding and clarifies any points that are unclear.

Will the findings of the research be made available in an understandable format to stakeholders in the community where the study was conducted (e.g., via a presentation, summary report, copies of publications, etc.)? Please provide details of how this will be achieved.

The findings of the research will be disseminated to stakeholders in multiple ways. First, the findings will be posted to the SES website, Facebook page, and X (formerly known as Twitter) account. These posts will be written in plain (non-scientific language) for the general public. SES has a full-time communications team that produces these and other similar materials. Next, Dr. Chiang, Dr. Lecca, and/or the study coordinator (Ms. Roman Sinche) will present the findings to the National TB Program Coordinator, as well as at an upcoming scientific meeting on TB held by the Peruvian Ministry of Health and attended by TB providers (physicians, nurses, nurse technicians). Finally, the publication of this article in an open access journal also will facilitate dissemination to Peruvian Ministry of Health officials and TB providers.
