# Supplementary figures and images for: Identifying adolescents at risk for suboptimal adherence to tuberculosis treatment: A prospective cohort study
Source: PLOS Glob Public Health. 2024 Feb 27;4(2):e0002918. doi: 10.1371/journal.pgph.0002918 (PMC10898721; doi:10.1371/journal.pgph.0002918)

**S1 Fig: Psychological care algorithm**

*
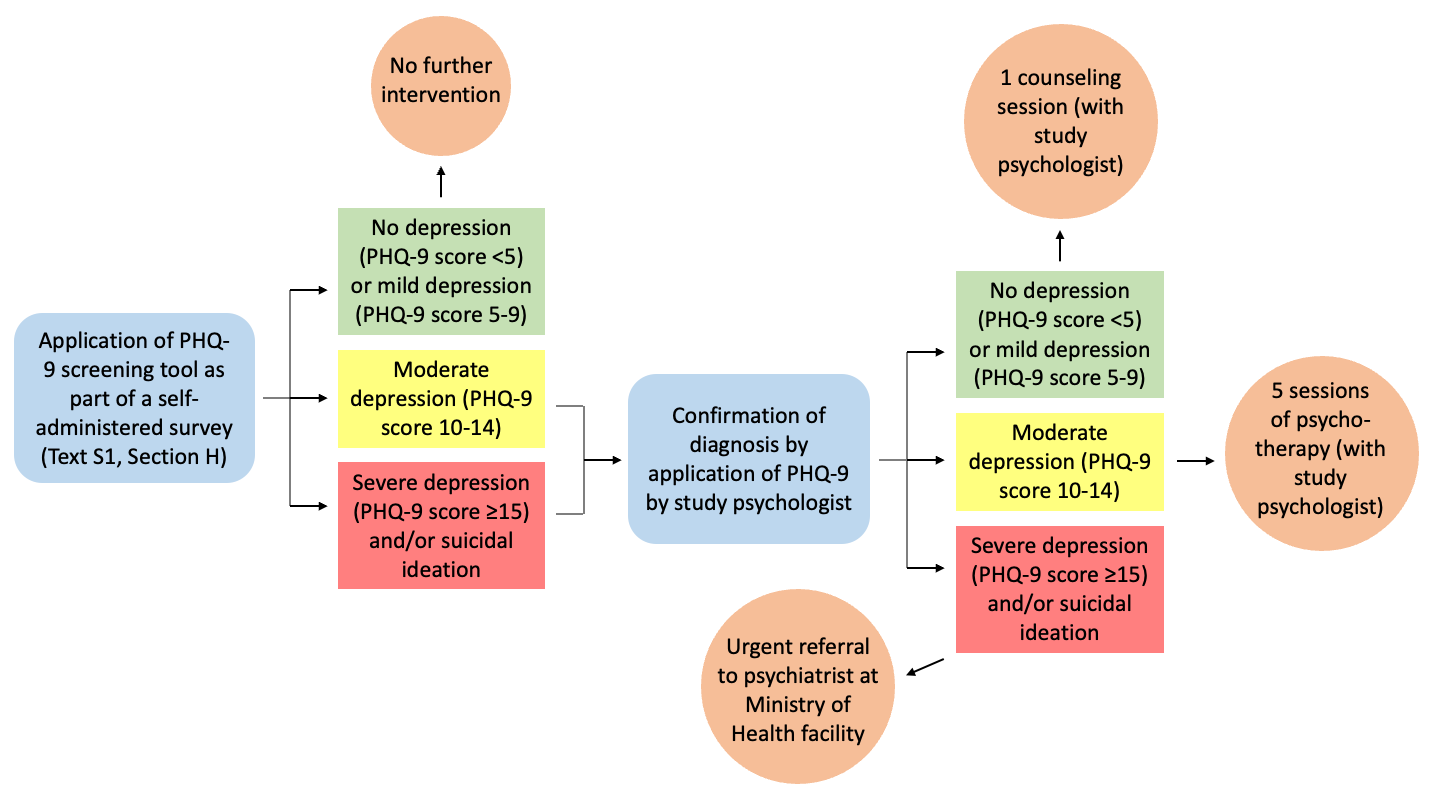
*

Supplement: S1 Fig — (DOCX) [file pgph.0002918.s002.docx]
