# Supplementary material for: Identifying adolescents at risk for suboptimal adherence to tuberculosis treatment: A prospective cohort study
Source: PLOS Glob Public Health. 2024 Feb 27;4(2):e0002918. doi: 10.1371/journal.pgph.0002918 (PMC10898721; doi:10.1371/journal.pgph.0002918)
Supplement: S1 Table — (DOCX) [file pgph.0002918.s003.docx]

**S1 Table: Demographic, psychosocial, and clinical characteristics, stratified by treatment setting**

| **n (%) or median (IQR)** | **All regimens**  **(n = 266)** | **Facility-based regimens**  **(n = 164)** | **Home-based regimens**  **(n = 102)** | **p-Value^*^** |
| --- | --- | --- | --- | --- |
| Male gender | 169 (63.5) | 99 (60.4) | 70 (68.6) | 0.22 |
| Age (years) | 17 (15-18) | 17.5 (15-18) | 16.5 (15-18) | 0.08 |
| Travel time to health center (minutes)^&^ | 9 (3-9) | 9 (9-9) | 9 (3-9) | 0.02 |
| Walks/bikes to health center (vs. takes taxi/motor-taxi/bus) | 143 (53.8) | 96 (58.5) | 47 (46.1) | 0.06 |
| Frequency of hunger due to poverty (days/week)^&^ | 0 (0-1.8) | 0 (0-1.8) | 0 (0-1.8) | 0.35 |
| Lives with mother | 229 (86.1) | 136 (82.9) | 93 (91.2) | 0.09 |
| ACE score (range: 0-10) | 1 (0-3) | 1 (0-3) | 1 (0-2) | 0.31 |
| Caregiver support score: 5 (least) to 25 (most) | 23 (19-25) | 23 (19-25) | 23 (19-25) | 0.89 |
| Obeys caregiver: 1 (never) to 5 (always) | 4 (4-5) | 4 (4-5) | 4 (3-5) | 0.27 |
| Support from friends: 1 (least) to 5 (most) | 4 (2-5) | 4 (2-5) | 4 (2-5) | 0.64 |
| Support from other family members: 1 (least) to 5 (most) | 5 (3-5) | 5 (3-5) | 4 (3-5) | 0.65 |
| Frequency of being accompanied to the health center for treatment: 1 (least) to 5 (most) | 3 (2-5) | 3 (2-5) | 4.5 (3-5) | 0.007 |
| PHQ-9 depression scale (range: 0-27) | 8 (5-12) | 8 (5-12) | 8 (4.3-11) | 0.57 |
| AUDIT score (range: 0-40) | 0 (0-0) | 0 (0-0) | 0 (0-0) | 0.55 |
| Illicit drug use in last 12 months | 40 (15) | 27 (16.5) | 13 (12.7) | 0.52 |
| Motivation score: 4 (least) to 20 (most) | 20 (18-20) | 20 (19-20) | 19 (17-20) | 0.01 |
| Anticipated stigma of seen receiving TB care: 1 (never) to 5 (always) | 1 (1-2) | 1 (1-2) | 1 (1-2) | 0.76 |
| Frequency of adverse treatment events (days/week)^&^ | 1.75 (0-3.5) | 1.75 (0-3.5) | 0 (0-1.75) | 0.001 |
| Satisfaction with TB care: 4 (least) to 20 (most) | 16 (15-17) | 16 (16-17) | 16 (15-17) | 0.70 |
| Family member on concomitant TB treatment | 28 (10.5) | 17 (10.4) | 11 (10.8) | 1.00 |
| Extrapulmonary involvement | 29 (10.9) | 20 (12.2) | 9 (8.8) | 0.35 |
| Microbiological confirmation | 205 (77.1) | 126 (76.8) | 79 (77.5) | 1.00 |
| isoniazid-monoresistance | 27 (10.2) | 20 (12.2) | 7 (6.9) | 0.23 |
| Daily dosing in continuation phase | 27 (10.2) | 19 (11.6) | 8 (7.8) | 0.44 |
| Regimen includes FDCs | 86 (32.3) | 46 (28.0) | 40 (39.2) | 0.08 |

Abbreviations: ACE, adverse childhood experience; AUDIT, Alcohol Use Disorders Identification Test; FDC, fixed-dose combination; PHQ-9 (Patient Health Questionnaire-9); TB, tuberculosis.

*Reported p-values tested the assumption that the reported statistics (median or percentage) were equivalent between all three clusters. Because the groups were determined empirically by cluster analysis, we did not have an *a priori* hypothesis as to which groups would have higher or lower levels of each variable. This column is provided for the specific purpose of identifying which traits tended toward the largest differences between groups.

^&^In the original survey, travel time to health center, the frequency of hunger, and the frequency of adverse treatment events were categorial variables, with response options given as fixed ranges. The only response option that did not include a fixed range was >2 hours for travel time to health center, but no participants selected this option. To facilitate statistical analysis, we converted these variables from categorical to continuous by replacing each range with its midpoint value. For example, 0-4 minutes (the first option for travel time to health center) was replaced by 2 minutes.
