# Supplementary material for: Identifying adolescents at risk for suboptimal adherence to tuberculosis treatment: A prospective cohort study
Source: PLOS Glob Public Health. 2024 Feb 27;4(2):e0002918. doi: 10.1371/journal.pgph.0002918 (PMC10898721; doi:10.1371/journal.pgph.0002918)
Supplement: S2 Table — (DOCX) [file pgph.0002918.s004.docx]

**S2 Table: Covid-related variables, stratified by cluster**

| **Variables** | **Cluster A**  **(n = 116)** | **Cluster B**  **(n = 83)** | **Cluster C**  **(n = 50)** | **p-Value** |
| --- | --- | --- | --- | --- |
| Degree of worry about getting Covid-19 at the health center: 1 (not at all) to 4 (very) | 2 (1, 2) | 2 (1, 2) | 2 (1, 2) | 0.20 |
| Degree of worry about getting Covid-19 on the way to the health center: 1 (not at all) to 4 (very) | 2 (1, 2) | 2 (1, 2) | 2 (1, 2) | 0.11 |
