# Supplementary material for: Identifying adolescents at risk for suboptimal adherence to tuberculosis treatment: A prospective cohort study
Source: PLOS Glob Public Health. 2024 Feb 27;4(2):e0002918. doi: 10.1371/journal.pgph.0002918 (PMC10898721; doi:10.1371/journal.pgph.0002918)
Supplement: S3 Table — (DOCX) [file pgph.0002918.s005.docx]

**S3 Table: Full regression output for suboptimal dose-based adherence**

| **Fixed effects*** | **Estimate** | **Standard error** | **p-Value** |
| --- | --- | --- | --- |
| Cluster A, facility-based, single drug formulation | Ref | Ref | Ref |
| Cluster B, facility-based, single drug formulation | 0.67 | 0.80 | 0.40 |
| Cluster C, facility-based, single drug formulation | 1.76 | 0.74 | 0.02 |
| Cluster A, home-based, single drug formulation | -0.59 | 1.18 | 0.62 |
| Cluster B, home-based, single drug formulation | -2.41 | 1.55 x 10^5^ | 1.00 |
| Cluster C, home-based, single drug formulation | -0.76 | 1.63 | 0.64 |
| Cluster A, facility-based, fixed dose combination | -0.87 | 0.86 | 0.31 |
| Cluster B, facility-based, fixed dose combination | -1.50 | 1.44 | 0.30 |
| Cluster C, facility-based, fixed dose combination | -1.20 | 1.48 | 0.42 |
| Cluster A, home-based, fixed dose combination | -0.17 | 1.68 | 0.92 |
| Cluster B, home-based, fixed dose combination | -1.52 | 1.68 x 10^7^ | 1.00 |
| Cluster C, home-based, fixed dose combination | 0.96 | 2.55 | 0.71 |

*Random effects: participant: variance 0, standard deviation 0; health center: variance 0, standard deviation 0.
