# Supplementary material for: Identifying adolescents at risk for suboptimal adherence to tuberculosis treatment: A prospective cohort study
Source: PLOS Glob Public Health. 2024 Feb 27;4(2):e0002918. doi: 10.1371/journal.pgph.0002918 (PMC10898721; doi:10.1371/journal.pgph.0002918)
Supplement: S4 Table — (DOCX) [file pgph.0002918.s006.docx]

**S4 Table: Time-based adherence, stratified by treatment setting and drug formulation**

| **Cluster** | **Total regimens (N)** | **Percent of regimens with suboptimal adherence (95% CI)** | **OR (95% CI) of suboptimal adherence*** | **p-Value** |
| --- | --- | --- | --- | --- |
| Facility-based, single-drug formulation | | | | |
| A | 53 | 9.4 (4.0, 20.7) | Ref | Ref |
| B | 38 | 15.8 (7.3, 31.0) | 1.80 (0.51, 6.40) | 0.36 |
| C | 27 | 29.6 (15.6, 49.0) | 4.04 (1.17, 13.93) | 0.03 |
| Facility-based, fixed-dose combination | | | | |
| A | 24 | 16.7 (6.4, 36.9) | Ref | Ref |
| B | 17 | 5.9 (0.8, 32.0) | 0.31 (0.03, 3.08) | 0.32 |
| C | 5 | 0 (0, 0) | NA^#^ | NA^#^ |
| Home-based, single-drug formulation | | | | |
| A | 31 | 6.5 (1.6, 22.4) | Ref | Ref |
| B | 19 | 5.3 (0.7, 29.4) | 0.81 (0.07, 9.54) | 0.86 |
| C | 12 | 16.7 (4.2, 47.7) | 2.90 (0.36, 23.39) | 0.32 |
| Home-based, fixed-dose combination | | | | |
| A | 16 | 12.5 (3.1, 38.6) | Ref | Ref |
| B | 16 | 0 (0, 0) | NA^#^ | NA^#^ |
| C | 8 | 12.5 (1.7, 53.7) | 1.00 (0.08, 13.02) | 1.00 |

*Model contained drug formulation (single drug formulation vs. fixed dose combination) and treatment setting (facility-based vs. home-based) as interaction terms.

^#^Unable to estimate due to 0 observations of suboptimal adherence in that group.

Abbreviations: CI, confidence interval; NA, not applicable; OR, odds ratio.
