# Supplementary material for: Identifying adolescents at risk for suboptimal adherence to tuberculosis treatment: A prospective cohort study
Source: PLOS Glob Public Health. 2024 Feb 27;4(2):e0002918. doi: 10.1371/journal.pgph.0002918 (PMC10898721; doi:10.1371/journal.pgph.0002918)
Supplement: S5 Table — (DOCX) [file pgph.0002918.s007.docx]

**S5 Table: Full regression output for suboptimal time-based adherence**

| **Variables** | **Estimate** | **Standard error** | **p-Value** |
| --- | --- | --- | --- |
| Cluster A, facility-based, single drug formulation | Ref | Ref | Ref |
| Cluster B, facility-based, single drug formulation | 0.59 | 0.65 | 0.36 |
| Cluster C, facility-based, single drug formulation | 1.40 | 0.63 | 0.03 |
| Cluster A, home-based, single drug formulation | -0.41 | 0.87 | 0.64 |
| Cluster B, home-based, single drug formulation | -0.80 | 1.42 | 0.57 |
| Cluster C, home-based, single drug formulation | -0.33 | 1.24 | 0.79 |
| Cluster A, facility-based, fixed dose combination | 0.65 | 0.72 | 0.37 |
| Cluster B, facility-based, fixed dose combination | -1.75 | 1.34 | 0.19 |
| Cluster C, facility-based, fixed dose combination | -20.19 | 1.21 x 10^4^ | 1.00 |
| Cluster A, home-based, fixed dose combination | -0.08 | 1.28 | 0.95 |
| Cluster B, home-based, fixed dose combination | -1.67 x 10^3^ | 1.68 x 10^7^ | 1.00 |
| Cluster C, home-based, fixed dose combination | 19.12 | 1.20 x 10^4^ | 1.00 |

*Random effects: participant: variance 0, standard deviation 0; health center: variance 2.72 x10^-9^, standard deviation 5.22 x 10^-5^.
