# Supplementary material for: Identifying adolescents at risk for suboptimal adherence to tuberculosis treatment: A prospective cohort study
Source: PLOS Glob Public Health. 2024 Feb 27;4(2):e0002918. doi: 10.1371/journal.pgph.0002918 (PMC10898721; doi:10.1371/journal.pgph.0002918)
Supplement: S2 Text — (DOCX) [file pgph.0002918.s009.docx]

**S2 Text: Survey development, including scale validation**

We developed the survey based on findings from our previous qualitative study on adolescent adherence to TB treatment.^13^ We included the following validated scales to measure relevant variables: PHQ-9 (depression), AUDIT (alcohol use disorder), and ACEs questionnaire (adverse childhood experiences). We adapted and validated the Van Rie Stigma Scale, as elaborated in our recent publication.^22,23^ However, in this analysis, we considered only one item from the scale, the fear of being seen receiving TB treatment at the health center, as this was the component of TB-related stigma that was most related to adherence in our qualitative study.

Next, we refined the survey through one-on-one cognitive interviews with 45 adolescents who had completed treatment for rifampicin-susceptible TB disease in Lima in the preceding 12 months. We carried out nine rounds of five cognitive interviews each. After each round, the research team met to refine the questionnaire, with the goal of optimizing comprehension by our target population while providing the desired information.

We then performed a brief validity analysis of a caregiver support scale (8 items), the TB knowledge scale (3 items), the motivation scale (4 items), and the health services satisfaction scale (6 items). We used factor analysis methodology to evaluate internal structure validity and estimate reliability by McDonald’s coefficient $\omega$ (similar to Cronbach’s $\alpha$ though a better estimate of reliability [1]). We anticipated each measure would be unidimensional with loadings of similar magnitude; we evaluated this empirically.

All measures demonstrated unidimensionality on the scree plot, acceptable reliability (i.e., $\hat{\omega}$ > 0.75), and reasonably sized positive loadings (i.e., $\hat{\lambda}$ > 0.3 [2]). Additionally, increasing the factors extracted beyond one never indicated subsets of questions with simple structure to the loadings that could constitute further subscales. That said, even if no items showed enough discriminating variation to be considered a subscale, some changes were made to improve content validity.

Three items were removed from the caregiver support scale (S1 Text, Section C). The questions all had proportionally smaller loadings than other items and represented how family members interact with others outside of the family. This simplified the internal structure and supported this as a measure of satisfaction with the family caregiver dynamics. Most patients being cared for by family members, this seemed to be a more relevant measure. Reliability was strong ($\hat{\omega}$ = 0.90, 95% CI: 0.86, 0.93).

The TB knowledge scale (S1 Text, Section F) also demonstrated unidimensionality and appropriate reliability ($\hat{\omega}$ = 0.76, 95% CI: 0.70, 0.80). However, the question that we thought was most important had the smallest loading ($\hat{\lambda}$ = 0.34, other loadings > 0.5 ). For this reason, we elected to focus on this single question, representing awareness of the risks of missed doses. While we may not have been able to estimate reliability for this single item, it was correlated with other items on the same scale, and this decision improved content validity.

No changes were made to the motivation scale (S1 Text, Section E). The pattern of loadings did not indicate systematic differences in measurement, and reliability was good ($\hat{\omega}$ = 0.85, 95% CI: 0.80, 0.89).

Lastly, three items were removed from the health services satisfaction scale (S1 Text, Section D). While the loadings on these were in some cases large ($\hat{\lambda}$ > 0.65), these items all represented facility characteristics, including satisfaction with the facility space and hours of availability. We elected to focus on a subset of items emphasizing how participants felt healthcare providers listened to concerns, respected them individually, and explained their condition. This change still had appropriate reliability ($\hat{\omega}$ = 0.77, 95% CI: 0.72, 0.82).

References:

1. Dunn TJ, Baguley T, Brunsden V. (From alpha to omega: A practical solution to the pervasive problem of internal consistency estimation. Br J Psychol 2014;105(3), 399-412.
2. Harlow, LL. The essence of multivariate thinking: Basic themes and methods, 2^nd^ d. New York, USA: Routledge/Taylor & Francis Group, 2014.
