# Supplementary material for: Identifying adolescents at risk for suboptimal adherence to tuberculosis treatment: A prospective cohort study
Source: PLOS Glob Public Health. 2024 Feb 27;4(2):e0002918. doi: 10.1371/journal.pgph.0002918 (PMC10898721; doi:10.1371/journal.pgph.0002918)
Supplement: S3 Text — (DOCX) [file pgph.0002918.s010.docx]

**S3 Text: Sample size and power**

Due to higher project costs and less access to potential participants during the COVID-19 pandemic, we were only able to collect data on 249 participants. A secondary power analysis was performed to evaluate whether or not the sample obtained was still sufficient to detect meaningful differences. The analysis was formulated around groups empirically identified by cluster analysis, so, to estimate what might be a lower limit on power, we considered power for the smallest groups, with 50 and 83 participants in each group. First, we considered power to detect a difference between these groups. Assuming the null suboptimal treatment adherence at 20% (Chiang SS, et al. Int J Tuberc Lung Dis. 2020;24(7):723-725), we estimated that we would need to observe an odds ratio of 2.50.

We were also interested in evaluating whether or not treatment adherence changed over time. Power to detect an association for the smallest group, with only 50 participants, was estimated. The distribution of treatment duration was assumed to be exponential to allow for the possibility of skew that would reduce power, i.e., if most participants had relatively short follow-up times and relatively few followed up in the last months of treatment. While it is unlikely that the average follow-up time would be one month, we assumed the rate parameter was one month so that the variance was equal to one, thereby removing the mathematical artifact of scaling so that it can be interpreted as a standardized variable while accounting for possible skew. Power under these conditions suggested that we were able to detect an odds ratio of 3.01.

Taken together, these secondary power calculations suggests that we likely had sufficient power to detect moderate associations and differences for the smallest groups, and that power was likely even better for larger groups and comparisons. Additionally, one of the reasons we used cluster analysis to identify groups was for feature selection. Cluster analysis can result in the identification of small groups and dichotomization of continuous measures, both of which risk reducing power analytically. However, because cluster identities are based on maximizing differences between groups and were reviewed for clinical relevance prior to including in the analysis, this approach can help to identify groups anticipated to have larger differences. The loss to power in forming potentially small groups is anticipated to come with the benefit of larger effect sizes between groups.
